# Insular thyroid carcinoma in a young Moroccan man: Case report and review of the literature

**DOI:** 10.1016/j.amsu.2022.103592

**Published:** 2022-04-06

**Authors:** Wahiba Abdellaoui, Imane Assarrar, Salma Benyakhlef, Abir Tahri, Najoua Messaoudi, Anass Haloui, Siham Rouf, Amal Bennani, Hanane Latrech

**Affiliations:** a Department of Endocrinology-Diabetology-Nutrition, Mohammed VI University Hospital, Medical School, Mohammed the First University, Oujda, Morocco; b Anathomopathology Laboratory, Mohammed VI University Hospital, Medical School, Mohammed the First University, Oujda, Morocco; c Laboratory of Epidemiology, Clinical Research and Public Health, Faculty of Medicine and Pharmacy of Oujda, Mohammed the First University, Morocco

**Keywords:** Case report, Insular thyroid carcinoma, Thyroid cancer, PDTC, Poorly differentiated thyroid carcinoma, CMIA, Chemiluminescence Microparticle Immunoassay

## Abstract

**Introduction:**

Insular thyroid carcinoma (ITC) was defined as a rare malignant thyroid cancer standing in an intermediate position between the well-differentiated (papillary and follicular) and the anaplastic thyroid carcinomas. The incidence was estimated around <1% and 10% worldwide. Despite its rarity, it remains the main cause of death from non-anaplastic follicular cell-derived thyroid cancers.

**Case presentation:**

A 27-year-old single male admitted for a history of a thyroid nodule and intrathoracic extension; with local mass effect, deviating the brachiocephalic trunk to the right. He underwent a total thyroidectomy. Histopathological examination showed a poorly differentiated insular thyroid carcinoma. Radioactive iodine-131 therapy was administred at a dose of 100 mCi, and the patient was maintained on TSH-suppressive therapy. Ultrasensitive Thyroglobulin measurement after thyroxine withdrawal, taken 2 years after radioactive iodine treatment was undetectable as well as thyroid antithyroglobulin antibodies.

**Conclusion:**

Our clinical case would enrich the global registry of insular thyroid carcinomas’ cases. The main challenge is early detection, aggressive intervention, and close follow-up of affected patients. The advancement in ultra-deep sequencing technologies, will contribute in the development of novel targeted therapies aiming to reduce morbidity and mortality and improve the outcomes in PDTC patients as well.

## Highlights

• Poorly differentiated thyroid carcinomas are defined according to insular growth features, increased mitotic rate, and aggressive clinical behavior.
• Insular thyroid carcinoma is the main cause of death from non-anaplastic follicular cell-derived thyroid cancers.

## 1 Introduction

Insular thyroid carcinoma (ITC) was described as a rare malignant thyroid cancer standing in an intermediate position between the well-differentiated (papillary and follicular) and the anaplastic thyroid carcinomas; incorporated in 2004 in World Health Organization classification of poorly differentiated thyroid carcinomas. The incidence was estimated around <1% and 10% worldwide. A higher frequency was noticed in some countries like Italy but it was less common in USA. This tumor concerns predominantly women aged between 44 and 66 years [1]. Particular criteria which define poorly differentiated thyroid carcinomas are still arguable, but include mostly: insular growth features, increased mitotic rate, and aggressive clinical behavior [2].

Despite its rarity, it remains the main cause of death from non-anaplastic follicular cell-derived thyroid cancers, and is then highly significant. Data about this tumors' prognosis and outcome is limited, and correlation with non-insular, differentiated thyroid carcinomas wasn't assessed enough in litterature. Nevertheless, the poorer outcome was related to distant metastases, the rate of persistent disease, and disease specific death [3]. The mean rate of recurrence or metastases noted in the literature ranges from 36% to 83% and the disease-specific mortality from 9% to 75%.

In this paper, we consider a new case of a young 27 year-old man presented with insular thyroid carcinoma without capsular invasion or distant metastases. We discuss as well, its diagnosis, behavior and therapeutic approaches besides, our literature review highlights. To our knowledge, this is the first young Moroccan case reported in the literature. This case has been reported following the SCARE criteria [4].

## 2 Case report

A 27-year-old single male presented with a right cervical mass. He first consulted a general surgeon since the mass was growing rapidly. The patient was a chronic smoker. He didn't receive any radiation therapy and didn't mention any history of thyroid disease or malignant neoplasm in the family, however the patient comes from a mountainous area. He was clinically euthyroid; without flushing disorder nor obstructive symptoms. The physical examination revealed a ferm well-defined right thyroid nodule, with no cervical lymphadenopathies and a negative Pemberton's sign. Thyroid ultrasound revealed a well circumscribed heterogeneous nodule of the right lobe measuring: 30*27*19 mm; containing both solid and cystic components with intracystic vegetations. The computed tomography scan of the neck exhibited an enlarged right thyroid lobe with intrathoracic extension; measuring 48*33*23 mm and having both solid and fluid elements; causing local mass effect, deviating the brachiocephalic trunk to the right.

Thyroid function tests and calcitonin were within normal range: TSUus: 1,36 mUI/l (0,27-4,2; CMIA), FT4: 16,3 pmol/l (12–22; CMIA), Calcitonin: 0,68 pg/ml (8,31-14,3; CMIA); excluding the possibility of a medullary thyroid carcinoma.

The patient underwent a right hemithyroidectomy without any postoperative complications. Histological examination described, poorly differentiated insular thyroid carcinoma of 4cm which is unifocal without neither capsular invasion nor tumor emboli; classified: pT2NxMX according to AJCC 8th edition/TNM classification system. Then, a total thyroidectomy was performed without previous fine-needle aspiration. No signs of malignancy have been described in the left lobe. Radioactive iodine-131 therapy was administred at a dose of 100 mCi. The I-131 whole body scan performed 7 days after the ablative treatment, detected 4 median foci of intense uptake in the thyroid bed. TSH-suppressive therapy was initiated at a dose of 2 μg/kg/day; and the patient was referred to our department for further exploration and management. However, it was necessary to confirm the insular histological features, before making decisions either on therapeutic pathway or adequate follow-up.

**Fig. 1:**
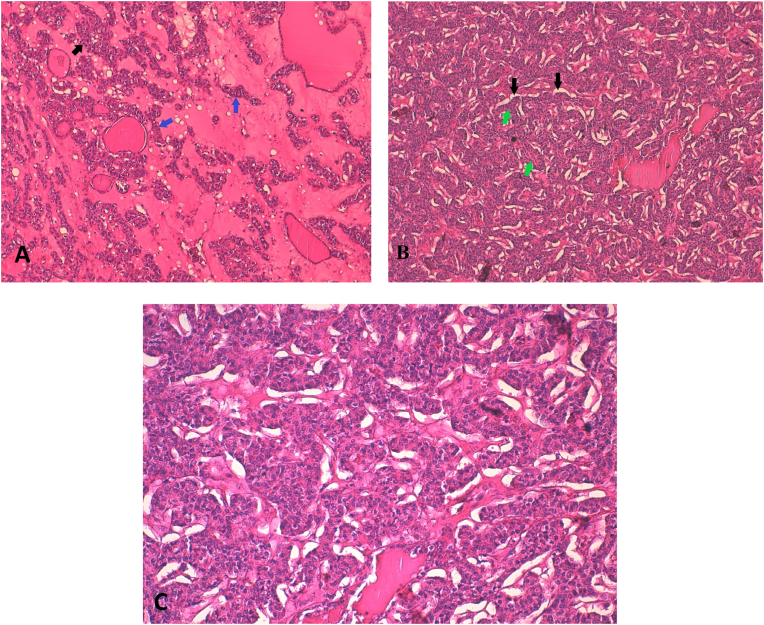
Microphotography of the specimens. **A:** Thyroid follicles dissociated by a carcinomatous tumoral proliferation of solid (black arrow) and insular architecture (blue arrows) (LPF, 10×Magnification); **B:** Tumor clumps (green arrows) are surrounded by cleft-like spaces (black arrows) (20x Magnification); **C:** The tumoral cells are provided with irregular nuclei, showing moderate to severe atypia, and an abundant eosinophilic cytoplasmic.Numerous mitotic figures can be identified, Absence of nuclear criteria for papillary carcinoma.The tumor cells express Thyroglobuline, whereas the Calcitonin is negative (40x Magnification).

The final histopathological examination described thyroid follicles dissociated by a carcinomatous tumoral proliferation of solid and insular architecture. Tumor clumps were surrounded by cell-like spaces. Tumor cells had irregular nuclei showing moderate to severe atypia with numerous mitotic figures, besides the absence of necrosis and nuclear criteria for papillary carcinoma. Immunohistochemical staining was positive for Thyroglobulin, and negative for Calcitonin (Fig. 1).

The patient was maintained on TSH-suppressive therapy. Ultrasensitive Thyroglobulin measurement after thyroxine withdrawal, taken 2 years after radioactive iodine treatment was undetectable as well as thyroid antithyroglobulin antibodies. The patient was on regular follow-up and is still clinically well 3 years after the diagnosis.

## 3 Discussion

An insular variant of thyroid carcinomas was singled out by Langhans in 1907, and in 1984, Carcangiu et al. reported for the first time its pathognomonic histological findings as well-defined nests or « insulae » of uniformly shaped cells; in a group of 25 patients with poorly differentiated thyroid carcinomas (PDTC) [5]. We collected and summarized available literature data on insular carcinoma in Table 1. In literature, this tumor concerned predominantly women aged between 44 and 66 years [1]. Our patient, was a male nearly two decades younger (27 years), which is not commonly seen in literature.

**Table 1:** Characteristics of published cases and series in literature.

Patient **(n)**	Sex (F/M)	Mean age	Recurrence/Metastasis **(n)**	Mortality **(n)**	Total Thyroidectomy	Radioactive Iodine **(n)**	Author	Year
**25**	17/8	55	21	14	20	7	Carcangiu et al. [5]	1984
**4**	4/0	55	3	3	3	3	Flynn et al. [20]	1988
**2**	2/0	66	1	1	1	1	Killeen et al. [21]	1990
**5**	4/1	49	4	0	3	3	Justin et al. [22]	1991
**6**	4/2	32	2	2	6	6	Rodriguez et al. [1]	1998
**2**	2/0	50	2	1	2	2	Gómez et al. [23]	2005
**1**	1/0	65	0	0	1	0	Salih et al. [24]	2016
**1**	1/0	55	1	0	1	0	Saad et al. [25]	2018
**1**	0/1	55	1	0	1	1	Uçmak et al. [26]	2019
**1**	0/1	27	0	0	1	1	Abdellaoui et al.,	2022

Some of the common clinical signs are: long-standing goiter with dysphagia or dyspnea whenever the lesion is large. A history of a prior irradiation or iode deficiency are considered as risk factors, and distant metastases are noticed in 20% of cases as a revealing symptom. This rare entity is known for its aggressivity, and represents the major cause of death from non-anaplastic follicular cell-derived thyroid carcinomas. In general, lymph node metastases may be detected initially in 50–84,6% just like distant metastases especially in lungs and bones in 36,4 to 84,6%; but also in unusual sites like ovary, liver and skin [6]. Our patient presented initially with a thyroid nodule of the right lobe of about 4 cm, with local mass effect but without neither local nor distant metastases.

The diagnosis of ITC must be based on typical histologic patterns, which is usually strenuous for pathologists. This tumor is determined by well-defined nests (insulae) of small-sized and monomorphic neoplastic cells, thyroglobulin-containing follicles, necrotic foci and irregular but persistently mitotic-activity [7]; as it was described in our case.

Concomitant well-differentiated thyroid carcinomas were noted in insular carcinomas, and foci of insular carcinoma were also identified in anaplastic carcinomas. Recently, Setia et al. notified that, although atypical mitoses are detected in PDTC, they are less common than in anaplastic thyroid cancer [8].

Insular carcinomas display positive thyroglobulin immunostaining. Negative staining for cytoplasm immunoglobulin, calcitonin, and leukocyte common antigen helps distinguishing between insular carcinomas and malignant lymphomas or medullary cancers. Asioli et al. requested the inclusion of a new prognostic factor in PDTC, particularly, insulin-like growth factor-II mRNA-binding protein 3 (IMP3) expression, which can be assessed by immunohistochemistry [9]. Our case's results are pictured in (Fig. 1).

Regarding the worse prognosis of insular carcinomas, optimal treatment must be tailored by a multidisciplinary team. Total thyroidectomy with nodal resection for lymph node disease is the cornerstone of the treatment approach, even with distant metastases. Wide resection is correlated to low recurrence and low mortality rates [10]. Radioiodine uptake ability either of primary tumor or distant metastases exceeds 80%, which is much higher in patients who were treated right away after thyroidectomy compared to those who were treated after local recurrence or distant disease [11]. The impact of radioiodine treatment on the prognosis of patients with insular carcinoma is not well established. In a recent trial, clinical benefit from radioiodine therapy was denoted only in patients who didn't have TSH-R gene mutation and a remarkably high radioiodine uptake [3]. It was advocated that the radioiodine uptake is related to the efficiency of therapy response and a considerable prognostic factor for survival [3].

In patients with radioiodine avid tumors, high dose sequential radioiodine treatments with dosimetric studies, have to be applied concomitant with other treatment approaches. For iodine refractory tumors, risk-adapted customized molecular targeted therapies must be studied. Our patient was treated with 100 mCi of 131I, and received thyrotropin suppression therapy during three years with undetectable ultrasensitive thyroglobulin and negative anti-thyroglobulin antibodies.

The place of postoperative external beam radiation therapy (EBRT) in PDTC is still arguable, but it has been recommended in patients with T3, T4 tumors and in cases with neck node involvement [12]. Nonetheless, no significant survival enhancement has been documented in these cases after EBRT [13]. Reports have been scarce regarding chemotherapy in PDTC. There is level III evidence as reported by Sackett et al. with short follow-up, that patients with inoperable PDTC who underwent chemotherapy regimen with or without EBRT became operable or were free of disease [14]. In our case, neither EBRT nor chemotherapy were needed.

Published data relating this very rare subtype of thyroid carcinomas was limited to case reports, small institution series, and collected reviews [3], and comparison with non-insular, differentiated thyroid carcinomas wasn't studied enough in litterature. Up to date, there are two eminent and largest reports in literature. In 2012, Kazaure et al. summarized the SEER database experience with ITC from 1999 to 2007 and identified 114 patients [15]. The mean age was 62.1 years, average tumor size was about 5.9 cm, and the rate of distant metastasis didn't exceed 31%. Three years later, Pezzi et al. selected 508 patients from the NCDB (National Cancer Data Base), with data from patients treated at Commission on Cancer-approved programs during 15 years, and confirmed that ICT is a specific subtype of thyroid cancer with significant differences from anaplastic thyroid cancers and well-differentiated ones. In this study, only 20.8% of patients had distant metastases. However, around 60% of patients presented with advanced stage disease. Patients with ITC compared to FTC and PTC, were older, with larger tumors, and presented more frequently with distant metastases, but were less likely to achieve an R0 resection, and had unfortunately significantly lower survival [16]. Our patient was young, and is still clinically well 3 years after the diagnosis.

Insular thyroid carcinomas show a more aggressive course compared to DTC, with a higher predisposition for local recurrence. Nonetheless, it is not lethal as anaplastic and has a 62–85% 5-year overall survival [14] and a 66% 5-year disease free survival [14]. Recent studies report satisfying 5-year locoregional control in PDTC patients, if all gross disease is achieved at initial surgery. Otherwise, distant disease constitutes the main cause of death in PDTC, accounting for more than 85% of disease related deaths [14]. This is particularly crucial since treatment approaches have not been advantageous in preventing systemic spread of the disease. Then, the development of advanced targeted therapies is mandatory to improve outcomes.

Number of groups have published genomic findings in PDTC based on next-generation sequencing (NGS) techniques, in order to elucidate the molecular characteristics of PDTC which are liable for driving disease progression [17].

The most usual mutations in PDTC are *TERT* promoter mutations (40%) and the most commonly mutated tumor suppressor gene is *TP53* (16%). Takeuchi et al. identified mutations of the p53 gene in 38% of patients with ITC [18]. Nevertheless, mutually exclusive mutations of *BRAF* and *RAS* keep to be the most common driver mutations in PDTC, just like DTC and ATC [19].

Moreover, mutations of *EIF1AX*, *MED12* and *RBM10* have been recently described in PDTC and may predict a poor survival. Fatal PDTC compared to nonfatal PDTC exhibited a higher frequency of mutations in the *TERT* promoter, *MED12*, *RBM10*, *BRAF, HRAS*, *TP53*, *ATM* and *EIF1AX* [*14*]. In this new era of advanced generation of sequencing technologies, novel precision therapies will be tailored continuously, aiming to reduce morbidity and mortality in PDTC patients. Genetic tests were not performed in our case due to limited ressources.

## 4 Conclusion

Insular thyroid carcinoma is a rare clinical entity and the main challenge is early detection, aggressive intervention, and close follow-up of affected patients. The patients must undergo aggressive treatment involving total thyroidectomy, high-dose RAI, and adequate lymph node dissections [15].

Regarding the rarity of this tumor, and other poorly differentiated thyroid carcinomas, multi-institutional studies must be conducted in order to elaborate operational guidelines.

The recent insights into the clinicopathologic and molecular features of PDTC, with the advancement in ultra-deep sequencing technologies, will contribute in the development of novel targeted therapies aiming to reduce morbidity and mortality and improve the outcomes in PDTC patients as well [14].

## Sources of funding

This research was not funded.

## Provenance and peer review

Not commissioned, externally peer reviewed.

## Ethical approval

This is a case report that does not require a formal ethical committee approval. Data were anonymously registered in our database. Access to data was approved by the head of the department.

## Author contribution

Dr. Wahiba Abdellaoui wrote the manuscript.

Dr. Imane Assarrar helped in writing and literature review.

Dr. Salma Benyakhlef helped in writing and literature review.

Dr Abir Tahri helped in writing and literature review.

Dr. Najoua Messaoudi helped in writing and literature review.

Dr. Anass Haloui interpreted and provided pathological data.

Pr. Amal Bennani confirmed the histological diagnosis and revised the final manuscript.

Pr. Siham Rouf helped in writing, supervised the redaction and revised the manuscript.

Pr. Hanane Latrech helped in writing, supervised the redaction, revised and approved the final draft for publication.

All authors approved the final version of the manuscript.

## Consent

A written informed consent was obtained from the patient for publication of this case report and accompanying images. A copy of the written consent is available for review by the Editor-in-Chief of this journal on request.

## Registration of research studies

This is not an interventional study. We only reported the patient's findings from our database as a case report.

## Guarantor

Professor Hanane Latrech.

## Declaration of competing interest

We have no Competing interests about this scientifical article.
